# Intra-abdominal desmoid tumors in familial adenomatous polyposis: How much do clinical and surgical variables interfere with their development?

**DOI:** 10.1016/j.clinsp.2022.100144

**Published:** 2022-12-05

**Authors:** Fábio Guilherme Campos, Carlos Augusto Real Martinez, Leonardo Alfonso Bustamante-Lopez, Roberta Laís da Silva Mendonça, Danillo Toshio Kanno

**Affiliations:** a Hospital das Clínicas da Faculdade de Medicina da Universidade de São Paulo (HCFMUSP), São Paulo, SP, Brazil; b Universidade São Francisco (USF), São Paulo, SP, Brazil; c AdventHealth, Orlando, United States

**Keywords:** Desmoid tumors, Familial adenomatous polyposis, Ileal pouch-anal anastomosis, Ileorectal anastomosis, Risk factor, Laparoscopic surgery, Hereditary disease

## Abstract

**Objective:**

Familial Adenomatous Polyposis is a complex hereditary disease that exposes the carrier to a great risk of Colorectal Cancer (CRC). After prophylactic surgery, intra-abdominal desmoid tumors are known to be one the most important cause of death. Therefore, recognition of increased-risk patients and modification of operative strategy may be crucial.

**Aim:**

The objective of this study was to estimate the desmoid tumor risk in relation to various surgical and clinical variables.

**Methods:**

Patients who had undergone polyposis since 1958 were included in the study. After exclusion criteria were met, those who had developed desmoid tumors were selected to undergo further evaluation.

**Results:**

The study revealed that the risk of developing desmoid tumors was associated with various factors such as sex ratio, colectomy, and reoperations. On the other hand, the type of surgery, family history, and surgical approach did not affect the risk of developing desmoid tumors. The data collected from 146 polyposis patients revealed that 16% had desmoid polyps. The sex ratio was 7:1, and the median age at colectomy was 28.6 years. Family history, multiple abdominal operations, and reoperations were some of the characteristics that were common in desmoid patients.

**Conclusion:**

: Recognition of clinical (female sex) and surgical (timing of surgery and previous reoperations) data as unfavorable variables associated with greater risk may be useful during the decision-making process.

## Highlights

• Desmoid Tumors in Familial Adenomatous Polyposis are a huge problem in these patients, the ability to prevent desmoid development is limited.
• Some clinical and surgical variables were identified as unfavorable and associated with a greater risk.
• This variable may be useful during the decision-making process.

## Introduction

A complex genetic disorder known as Familial Adenomatous Polyposis (FAP) is linked to the growth of several colorectal adenomatous polyps with a high risk of malignancy. The disease's penetrance and manifestation make FAP the most recognizable hereditary colorectal syndrome.^1^

There are three different surgical options for FAP patients: total Proctocolectomy with end Ileostomy (PEI), total proctocolectomy with ileal pouch-anal anastomosis, and total colectomy and Ileo-Rectal Anastomosis (IRA) (RPC). Age, polyp load, desire for conception, health status, and other factors all influence surgical choice. Prophylactic colectomy is typically recommended between the ages of 20 and 25, as early surgery prior to that age is typically not necessarily due to the minimal risk of cancer at that time (1%).^2^^,^^3^ Since prophylactic colectomy reduces Colorectal Cancer (CRC) incidence, many extra-colonic manifestations of the disease become more clinically apparent.

FAP-associated Desmoid Tumors (DT) are recognized as a great challenge and the most disabling FAP extra-intestinal manifestation, being reported in approximately 15% of patients during their lifetime.^4^ Although incidental de novo lesions may be found at the primary surgery in about 3%‒4% of cases,^5^, ^6^, ^7^ 70%‒83% of them develop in a time lag of 2‒3 years after surgery, the reason why they have been linked to surgical trauma and fibroblasts-mediated tissue repair.^4^^,^^8^^,^^9^

Intra-Abdominal Desmoid Tumors (IADT) may be aggressive and infiltrative tumors that lack the ability to spread metastatically and might eventually cause serious morbidity (bowel obstruction, perforation, ureteric compression, and major vascular lesions), among other things.^10^^,^^11^ The primary treatment choices for DT multimodal management are surgery, non-steroidal anti-inflammatory medications, hormone therapy, and chemotherapy.^12^ Female gender, positive family history, genotype (mutations in APC, CTNNB1, and MUTYH genes^13^ and previous abdominal surgery have been incriminated as potential non-modifiable DT risk factors.^6^^,^^9^^,^^14^^,^^15^

There is a lot of debate on the best surgical method to reduce post-interventional desmoid growth; topics covered include the time of surgery, procedure type, abdominal approach, and others.^1^ Although many studies have noted an increased risk in patients receiving RPC due to mesentery tension,^16^^,^^17^ some studies have not found significant differences between the various procedures.^6^^,^^18^^,^^19^ On the other hand, new research has shown that RPC and laparoscopic surgery reduce the incidence of DT.^9^^,^^16^^,^^20^

The incidence and difficulties associated with DT therapy in a public tertiary hospital in Brazil have already been highlighted by the studied group.^10^ In this case, recognizing risk variables that could be involved in DT development is necessary for surgical planning for FAP patients.

## Materials and methods

The present manuscript was evaluated and approved by the Gastroenterology Department Ethics Committee in the *Hospital das Clínicas* (Memo CaPPesq 049/17).

Review of FAP patients undergoing surgery in a tertiary referral facility. The database at the institution was used to find these patients (retrospective 1958‒1995; prospective 1995‒2020). The development of intra-abdominal DT (with or without abdominal wall) after colectomy was the result that was of interest, whether it was seen clinically or radiologically. Patients were disqualified due to postoperative, desmoid, or surgical criteria.

Surgical causes included patients who had not undergone surgery, partial colectomies, internal deviations, or operations conducted during the previous two years. Desmoids on the abdominal wall or outside the abdomen were also disregarded. The exclusion group was made up of individuals who died suddenly fewer than 24 months following their colectomy. Study variables were selected based on literature data, trying to identify risk factors for DT.^9^^,^^15^^,^^21^^,^^22^ There were evaluated clinical (sex, age at first operation, age at DT diagnosis, family history of DT, diagnosis of colorectal cancer), surgical (interval from index surgery to DT, prophylactic or malignancy purpose, type of operative procedure, open or laparoscopic approach, previous abdominal operations, postoperative complications leading to abdominal sepsis and/or reoperation) and length of follow-up. As some previous studies^15^ suggested interesting risk groups by combining sex and age at colectomy, the authors also explored this idea.

Statistical analysis was performed trying to establish a correlation between clinical and surgical data with the development of DT. To analyze variables, the authors used Fisher's Exact Test. In order to compare results between two independent samples, the authors used a non-parametric test (Mann-Whitney). For both tests, the adopted significance level was 5% (p<0.05).

## Results

### Characteristics of patients with FAP

166 FAP patients were recorded in the hospital's database over a 62-year span. Twenty (20) patients were excluded from the analysis due to surgical exclusion (10), desmoid exclusion (5), or death (5) criteria, leaving 146 subjects for the study.

Clinical characteristics and surgical information for 146 operated patients are shown in Table 1. 64 (43.8%) males and 82 (56.2%) females were represented by the sexes. The median age at the time of FAP treatment was 33.7-years (11–82), with no gender differences. Excision of colorectal cancer was the primary surgical goal in 66 (45.2%) patients and preventative surgery in 80 patients (54.8%).

**Table 1:** Clinical characteristics and surgical data of 146 patients operated for FAP.

Variable	Number	(%)
Number of FAP patients	146	100%
Gender distribution		
Male	64	43.8%
Female	82	56.2%
Median age at treatment	33.7 years (11‒82)	
Male	34.9y (11‒67)	
Female	31.9y (13‒82)	
CRC diagnosis at surgery	66	45.2%
Surgical procedure		
IRA	56	38.3%
TPC	90	61.6%
Surgical approach		
Open	78	53.4%
Laparoscopic	68	46.5%
Surgical complications		
Open	14	17.9%
Laparoscopic	17	25.0%
Reoperations		
Open	6	7.7%
Laparoscopic	9	13.2%

FAP, Familial Adenomatous Polyposis; CRC, Colorectal Cancer; IRA, Total colectomy and Ileo-Rectal anastomosis; TPC, Total Proctocolectomy.

Total colectomy and Ileo-Rectal Anastomosis (IRA), Total Proctocolectomy (TPC) with Ileostomy (PCI) or with ileal-pouch anal anastomosis (RPC) were the surgical procedures used to treat the patients. IRA was the surgical choice in 56 (38.6%) patients, while total proctocolectomy was performed in 90 (61.6%).

Open (n = 78, 53.4%) or laparoscopic (n = 68, 46.5%) approaches were performed according to the surgeon's discretion. Reoperations were necessary for 15 (10.3%) patients, 6 (7.7%) after open and 9 (13.2%) after laparoscopic surgeries.

### Characteristics of desmoid tumor patients

Among the 146 selected patients, 16 (10.9%) were diagnosed with Intra-Abdominal Desmoid Tumors (IADT) during follow-up. Clinical features regarding this group are presented in Table 2. The sex ratio (F:M) was 7:1 (p = 0.018), and median ages were 28.6-years (17‒50) at colectomy and 31.9-years (19‒55) at DT diagnosis. The interval period between index surgery and DT was 30.7-months (14‒72).

**Table 2:** Clinical data of intra-abdominal desmoid disease patients.

Variable	Number	(%)
Intra-abdominal DT	16/146	10.9%
Distribution by gender (M/F)	Sex ratio = 1:7	[p = 0.018]
Median age at FAP treatment (range)	28.6 years (17‒50)	
Median age at DT diagnosis (range)	31.9 years (19‒55)	
Interval between surgery and DT diagnosis	30.7 months (14‒72)	
Family history of DT	5/16	31.2%
Association with CRC	7/16	43.7%
Multiple abdominal operations	9/16	56.2%
Reoperations after colectomy	5/16	31.2%

DT, Desmoid Tumor; CRC, Colorectal Cancer.

Family history (n = 5; 31.2%), association with CRC (n = 7; 43.7%), multiple abdominal operations (n = 9; 56.2%), and reoperations to treat complications after colectomy (n = 5; 31.2%) were frequent events among DT patients.

The association of DT rates with clinical and surgical data is presented in Table 3. Gender distribution showed that women were more likely to develop DT than males (female vs. male, 17% vs. 3.1%; p = 0.0074). When the authors confronted DT prevalence in relation to age at colectomy, the authors found no statistical difference when stratifying risk among patients with less than 19 years (14.8%), between 20‒29 years (15.8%), or older than 30 years (7.4%; p = 0.25). However, DT risk was greater (21.9% vs. 12.2%) among women who operated before 30 years (p = 0.023). Other variables such as surgical purpose (CRC excision vs. prophylactic, 10.6% vs. 11.2%, p = 0.88), type of surgical treatment (TPC vs. IRA, 12.2% vs. 8.9%, p = 0.59), surgical approach (Open vs. Laparoscopic, 10.2% vs. 11.8%, p = 0.79) and history of the desmoid disease (no history vs. positive history, 11.9% vs. 20.8%, p = 0.31) showed no statistical difference regarding DT risk. On the other side, reoperations for complications (no reoperation vs. reoperation, 8.4% vs. 33.3%, p = 0.01) influenced DT development.

**Table 3:** Desmoid tumor rates in relation to clinical and surgical variables.

Variables		Diagnosed DT (number)	%	p
Sex ratio				0.0074
Male	64	2	(3.1%)	
Female	82	14	(17%)	
Age at colectomy (16 DT/146 patients)				0.25
≤19 years	27	4	14.8%	
≤20‒29 years	38	6	15.8%	
≥30 years	81	6	7.4%	
Age at colectomy in female sex (14 DT/82 patients)				
≤19 years	19	3	15.8%	0.023
≤20‒29 years	22	6	27.3%	
≥30 years	41	5	12.2%	
Surgical purpose				1.00
Colorectal cancer excision	66	7	10.6%	
Prophylactic	80	9	11.2%	
Type of surgical treatment				
TPC (90) × IRA (56)		11 (12.2%) × 5 (8.9%)		0.72
Surgical approach				
Open (78) × Laparoscopic (68)		8 (10.2%) × 8 (11.8%)		0.97
History of desmoid disease (116 patients)				
No history (92) × positive history (24)		11 (11.9%) × 5 (20.8%)		0.31
Reoperations for complications				
No reoperation (131) × reoperation (15)		11 (8.4%) × 5 (33.3%)		0.01

DT, Desmoid Tumor; TPC, Total Proctocolectomy; IRA, Ileo-Rectal Anastomosis.

In Table 4, the authors compared DT rates in patients treated through open (n = 78) or laparoscopic (n = 68) approaches. There was no statistical difference in sex distribution, but those operated by laparoscopy were younger (30.8 vs. 35.7-years, p = 0.04) and had a shorter interval from surgery to desmoid disease (28.1 vs. 33.2-months, p = 0.04). DT rates according to the type of surgery and approach were not different either (p = 1.00).

**Table 4:** Clinical data and desmoid rates according to open or laparoscopic approaches.

Variables (entire series)	DT rate – Open surgery (n = 78)	DT rate – Laparoscopic (n = 68)	p
Sex ratio (M:F)			
Female (82)	40/78 (51.2%)	42/68 (61.7%)	
Male (64)	38/78 (48.7%)	26/68 (38.2%)	0.26
Median age at surgery	35.7 (11‒82)	30.8 (13‒75)	0.04
Interval surgery ‒ DT (months)	33.2 (20‒60)	28.1 (14‒72)	0.04
Follow-up (months)	70.3 (24‒288)	51.2 (24‒177)	0.001
Type of surgery and approach			
IRA (n = 56)	4/44 = 9.1%	1/12 = 8.3%	1.00
TPC (n = 90)	4/34 = 11.8%	7/56 = 12.5%	1.00

TPC, Total Proctocolectomy (with end ileostomy or pouch-anal anastomosis); IRA, Total colectomy with Ileo-Rectal Anastomosis; CRC, Colorectal Cancer; PO, Postoperative.

## Discussion

The majority of FAP patients will need surgery at some point in their lives, and the operational plan must consider factors including age, gender, polyposis burden, genotype, extracolonic symptoms, comorbidities, and individual characteristics.^1^, ^2^, ^3^ Both open and laparoscopic techniques to pouch surgery or IRA result in low morbidity rates and positive functional outcomes.^4^ Two to five years following surgery, one in six FAP patients may experience DT development. Surgery for DT is no longer the first-line treatment, except for emergency conditions (bleeding, organ obstruction, or perforation). It is quite challenging to completely remove mesenteric desmoids, and recurrence is common. Thus, a more conservative approach has been advised to reduce intestinal resection, risky surgery, and recurrence. At the same time, medical treatment limitations turn outcomes variable. Facing this scenario, most efforts have been directed toward preventing DT by eliminating or reducing modifiable risk factors.^5^

The intra-abdominal prevalence of FAP-associated desmoid illness and the stimulating effect produced by any operational method or approach has been highlighted in reports of the condition.^1^ In addition to the current manuscript, a few additional studies that focused solely on IAD also discovered a prevalence of 9%.^6^ Although the assessment of DT risk factors is debatable, prior abdominal surgery has consistently been linked to an increased chance of developing DT.^6^ Sinha et al. observed that those patients were three times more likely (OR = 3.35) to refuse abdominal surgery compared to others in a pooled analysis of 10 studies from 1965 and 2009.^7^ They confirmed in this analysis that 54% of DTs reported having had surgery previously. There are recommendations in this situation to put off surgery for individuals with favorable endoscopic characteristics.^8^^,^^9^

Among the DT patients, multiple abdominal operations (56%) and reoperations after index surgery (31%) were commonly reported. Moreover, reoperations in 15 patients were associated with a greater chance of desmoid disease (33% vs. 8.4%, p = 0.01). This fact reinforces the role of surgical trauma in DT physiopathology.

There are also many reports clearly indicating female sex is an independent clinical risk factor.^7^^,^^10^, ^11^, ^12^ In Canada, Durno et al.^8^ reported a 2.5 times greater chance in women who operated before 18 years of age (Hazard Ratio ‒ HR = 1.8), but a similar relation was not observed among men. Similarly, HR varying from 1.5 to 2.1 were reported in Italian^13^ and English^7^ studies. Consistently, the same idea was supported by a large series containing 2260 patients from 5 European countries.^14^ The present paper also identified a predominance in women (17% vs. 3%, p = 0.07), in whom DT risk was also different in three stratified age groups, as reported before among women undergoing surgery after 30 years of age.^8^

In the cohort of 16 IADT, the mean ages at colectomy and DT diagnosis were 28.6 and 32 years, respectively. The time between surgery and DT ranged from 14 to 72 months (median = 30.7). These figures are consistent with previously published data, which shows that DT occurrences range from 9% to 17% and that tumors are typically discovered between 2.5 and 5 years after surgery, at the beginning of the third decade.^14^^,^^15^ The authors demonstrated that DT incidence increases between the second and third decades of life in a beautiful meta-analysis involving 4625 individuals from ten studies.^6^

The present study's main result was to assess whether preventive colectomy led to the development of IADT (with or without the abdominal wall). By doing this, the authors were more interested in confirming how the extent of surgical trauma (by contrasting IRA and PCT) and the style of surgery (open or laparoscopic) could affect the development of desmoid illness in the abdominal cavity after surgery. To accomplish these goals, the authors excluded four patients who had extra-abdominal or abdominal wall desmoids exclusively, as well as those who had partial colectomies or palliative deviation surgeries. Finally, in light of the fact that the majority of DT would manifest 24 months after surgery,^6^^,^^7^^,^^13^^,^^16^ the authors additionally eliminated patients who had undergone surgery or who had died suddenly during the previous two years.

There are lots of controversies regarding which surgical strategy is more likely to predispose to DT.^10^^,^^17^ Taking into consideration that FAP patients are usually young, the option for a minimally invasive approach has been increasingly adopted. Safety and good outcomes are commonly reported advantages in many series,^18^, ^19^, ^20^ reviews,^21^ and comparative studies.^22^^,^^23^ Considering the less traumatic nature of laparoscopy, one preliminary idea is that it could influence DT risk favorably, as the small bowel and its mesentery would suffer less retraction during dissection and less exposition to temperature alterations than during open surgery.^6^

Besides the lack of standardization and treatment bias that may limit interpretation, it is possible to extract some ideas from single-centers^24^ and multicenter retrospective studies^10^^,^^13^^,^^15^^,^^25^ that addressed how the surgical approach could interfere with DT risk. This comparison identified only one study demonstrating a significant difference favoring laparoscopic surgery (4.3% vs. 13%, p = 0.04).^13^ Using multivariable hazards regression analysis, this Italian study found an HR of 6.84 comparing open and laparoscopic surgery. However, 57 out of 73 laparoscopic cases were performed in patients undergoing IRA.

In the current investigation, the authors did not detect any variations in DT rates (10.9% Open vs. 11.8% Lap) in connection to the approach. Although the open patients were older (35.7 vs. 30.8 years, p = 0.04) at the time of FAP treatment, this difference was insufficient to lower risks. However, the authors found that the laparoscopic group had a shorter time between the index surgery and DT (28.1% vs. 33.2 months, p = 0.04) in addition to the absence of other risk variables such as female preponderance. As previously mentioned, most IRA patients underwent laparotomies (78.5%), while the majority of TPC procedures were laparoscopic (62%).

Thus, this whole scenario suggests that the less invasive features of the laparoscopic approach don't provide enough protection against DT formation. However, an English observational study from St Mark's (1996‒2006) found a lower risk of laparoscopic ileorectal anastomosis (4% vs. 16%, p = 0.04) in a group of 112 patients.^26^

Another important debate is the potential influence regarding the extension of the procedure. The authors found only two multicenter studies favoring IRA over TPC. Recently, a multicenter Japanese database identified proctocolectomy as an independent risk factor for postoperative DT occurrence (HR = 2.2; p = 0.03), a result not reported before.^10^ Similarly, a lower risk after IRA was identified one year later in a national French database (12% vs. 25%, p = 0.02).^15^ On the other hand, other studies^13^^,^^24^^,^^27^ demonstrated no differences between RPC and IRA, including one multicenter study^14^ with 2260 patients and one meta-analysis containing 1260 patients.^28^ In a retrospective review including 558 FAP patients only with IADT.^6^ There was no difference in DT rates among patients undergoing RPC (3.8%) or IRA (5.1%). The authors also evaluated only IADT rates, to verify if surgical trauma extension could interfere. But patients undergoing TPC, or IRA were equally affected once more (12.2% vs. 8.9%).

Comparing the DT rates for each process and method individually is another way to tackle the issue. Data from single-center (like ours and the Cleveland Clinic) and multicentric studies are presented in Table 5.^4^^,^^13^ None of them provided statistical comparisons of methods and approaches, making it impossible to draw firm conclusions. According to the Cleveland Clinic study, DT development is more likely to occur when pelvic dissection is included.^24^ They believed that the ileal-pouch anastomosis caused mesenteric tension, a difficult-to-detect impact. However, this belief was disproved.^9^^,^^12^ However, if this stretching were truly significant, the authors would likely observe a DT preponderance following RPC, but the data do not support this.^13^^,^^14^^,^^27^^,^^28^ Moreover, their technique of minimally invasive pouch surgery was defined as “surgery performed via a Pfannenstiel incision, with or without laparoscopic colonic mobilization”. This definition turns difficult to define the real nature of the approach.

**Table 5:** Literature series comparing desmoid tumors Incidence in familial adenomatous patients according to surgery and approach.

Authors	Open IRA	LAP IRA	Open RPC	Lap RPC	p
Vogel et al. [17]	15.8%	3.8%	6.3%	46.2%	Open = Lap IRA; p = 0.29
					Open = Lap RPC; p = 0.04
Vitellaro et al. [16]	15.1%	3.6%	18.0%	6.7%	Open > Lap; p = 0.04
Konichi et al. [26]	4.8%	13%	19%	22%	Open = lap; p = 0.08
Present paper	9.1%	8.3%	11.8%	12.5%	Open = lap; p = 0.79

Lap, Laparoscopic; RPC, Restorative Proctocolectomy; IRA, Total colectomy with Ileo-Rectal Anastomosis.

As already mentioned, and criticized, the Italian series showed better results after laparoscopy.^13^ And the Japanese publication only suggests that TPC maybe affects patients more than IRA, besides the absence of statistical difference.^4^ The controversy presented here supports the idea that although DT formation may depend on a triggering factor due to tissue trauma,^29^ the underlying mechanism responsible for postoperative DT formation remains unclear. Apparently, this risk doesn't seem to be directly associated with a particular type of surgery or approach.

The idea of delaying prophylactic surgery aims to postpone this effect, especially within families either reporting DT cases or specific germline mutations such as 3’ APC, that are also associated with an attenuated phenotype.^30^ As most patients with severe phenotype will probably undergo an early RPC, and those with attenuated disease (3’end) will usually be treated by IRA at a later moment, the authors may understand why the type of surgery may not always influence DT risk.

The desmoid disease may be correlated with an APC mutation anywhere throughout the gene,^1^^,^^29^^,^^31^ but a distal 3’ APC mutation has been classically associated with increased propensity.^32^^,^^33^ Vitellaro et al.^13^ considered mutation distal to codon 1400 as an independent risk factor (with a 3.8 Hazard Ratio). In a systematic review focusing on genotype-DT associations, Slowick et al.^34^ found most mutations located in the 3’region (codons 1310 to 2011). Moreover, the 5’region (codons 543‒713) also demonstrated an elevated 2.0 HR. In a group of 323 FAP patients with 77 (24%) DT, the frequency of 3′ mutations was significantly greater in patients (p = 0.017) and families (p = 0.027) with the desmoid disease. Apart from mutations 3′ of codon 1399 or 1444, other reports failed to demonstrate more correlations between genotype and DT incidence^1^

Within this context, current knowledge indicates that the APC mutation site correlates more with desmoid disease severity rather than incidence. The relevance of site mutation seemed not to be evident, especially for intra-abdominal tumors.^5^^,^^14^^,^^29^^,^^35^ Mutations 3’ of codon 1399 are associated with higher incidence, greater penetrance, more symptomatic DT disease, worse stage, and lethality.^1^

As a tertiary public hospital, the authors don't perform molecular evaluation due to economic reasons. Independently of the APC-phenotype correlation, some believe that the existence of DT familial clustering may be more relevant.^12^^,^^29^^,^^31^ Evaluation of large FAP cohorts demonstrated that a first-degree relative with DT increases 7 to 9-fold the risk.^7^^,^^12^^,^^29^ In this series, besides a greater incidence of DT in patients referring history of DT (20.8% vs. 11.9%), this result didn't reach statistical significance (p = 0.3). Similar to previous studies that fail to indicate the influence of cancer environment on DT development, surgical purpose didn't interfere with this risk either in the series.^14^

All the data presented here reinforce the complexity and the challenging nature of the desmoid disease. As a particularly prone cohort, FAP patients may develop these tumors in varying incidences of 8%‒21%,^15^ although these numbers may increase if incidental (3%‒36%) lesions are included.^36^ During surgery, the discovery of two-dimensional lesions (named flat sheets, mesenteric plaque-like changes, desmoid reaction, and desmoid precursor lesion) may be a common event. Otherwise, the clinical significance and behavior of these lesions remain incompletely understood. Why and how many of them will turn into three-dimensional or mass lesions is a crucial question.

Several other facts may affect DT incidence. Surgical trauma, hormonal influences, and genotype have been evaluated in publications including data from Polyposis Registries, multicentric series, reviews, and meta-analyses. Different diagnostic criteria, patient features coming from referral centers, surgical technique, family history, and other non-modifiable variables are heterogeneous features that make comparison among different cohorts very difficult.

As a result, surgeons have little control over DT prevention. Evidently, the risk of IADT following surgery is unaffected by surgical decisions about approach and type of technique. Other than that, patient traits including sex, waiting too long to have surgery, and repeat surgeries tend to be adverse circumstances.

However, there is still a lot to learn about their biology, range of appearance, behavior, and, most importantly, the mechanisms causing their creation.

## Funding

There was no funding support involved in this manuscript.

## Ethics approval

The present manuscript was evaluated and approved by the Gastroenterology Department Ethics Committee in the Hospital das Clínicas (Memo CaPPesq 049/17).

Consent for publication (include appropriate statements): The authors consent to the publication of the present manuscript if it is accepted.

## Conflicts of interest

The authors declare no conflicts of interest.
